# Electrical impedance tomography during major open upper abdominal surgery: a pilot-study

**DOI:** 10.1186/1471-2253-14-51

**Published:** 2014-07-05

**Authors:** Maximilian S Schaefer, Viktoria Wania, Bea Bastin, Ursula Schmalz, Peter Kienbaum, Martin Beiderlinden, Tanja A Treschan

**Affiliations:** 1 Department of Anaesthesiology, Duesseldorf University Hospital, Heinrich-Heine University Duesseldorf, Moorenstraße 5, Duesseldorf 40225, Germany; 2 Department of Anaesthesiology, Marienhospital Osnabrück, Bischofsstraße 1, Osnabrück 49074, Germany

**Keywords:** Electrical impedance tomography, General anesthesia, Abdominal surgery, Atelectasis, Lung function

## Abstract

**Background:**

Electrical impedance tomography (EIT) of the lungs facilitates visualization of ventilation distribution during mechanical ventilation. Its intraoperative use could provide the basis for individual optimization of ventilator settings, especially in patients at risk for ventilation-perfusion mismatch and impaired gas exchange, such as patients undergoing major open upper abdominal surgery. EIT throughout major open upper abdominal surgery could encounter difficulties in belt positioning and signal quality. Thus, we conducted a pilot-study and tested whether EIT is feasible in patients undergoing major open upper abdominal surgery.

**Methods:**

Following institutional review board’s approval and written informed consent, we included patients scheduled for major open upper abdominal surgery of at least 3 hours duration. EIT measurements were conducted prior to intubation, at the time of skin incision, then hourly during surgery until shortly prior to extubation and after extubation. Number of successful intraoperative EIT measurements and reasons for failures were documented. From the valid measurements, a functional EIT image of changes in tidal impedance was generated for every time point. Regions of interest were defined as horizontal halves of the picture. Monitoring of ventilation distribution was assessed using the center of ventilation index, and also using the total and dorsal ventilated lung area. All parameter values prior to and post intubation as well as extubation were compared. A p < 0.05 was considered statistically significant.

**Results:**

A total of 120 intraoperative EIT measurements during major abdominal surgery lasting 4-13 hours were planned in 14 patients. The electrode belt was attached between the 2^nd^ and 4^th^ intercostal space. Consecutive valid measurements could be acquired in 13 patients (93%). 111 intraoperative measurements could be retrieved as planned (93%). Main obstacle was the contact of skin electrodes. Despite the high belt position, distribution of tidal volume showed a significant shift of ventilation towards ventral lung regions after intubation. This was reversed after weaning from mechanical ventilation.

**Conclusions:**

Despite a high belt position, monitoring of ventilation distribution is feasible in patients undergoing major open upper abdominal surgery lasting from 4 to 13 hours. Therefore, further interventional trials in order to optimize ventilatory management should be initiated.

## Background

Electrical impedance tomography (EIT) of the lung is a relatively new on-site tool to visualize and quantify intra-thoracic ventilation-dependent gas distribution [1]. EIT images represent changes in regional bio impedance. The images are derived from multiple skin electrodes positioned on the thoracic circumference [2]. Small alternating currents are applied to the electrodes and changes in conductivity of the underlying tissue are measured. The signals are processed and filtered and thus relative changes in impedance are a result of ventilation and tidal volume distribution in the lung. In patients undergoing mechanical ventilation EIT can be used to monitor recruitment maneuvers, to detect individual optimal positive end-expiratory pressure levels and to improve tidal volume distribution [3-5]. In clinical practice EIT is mostly used in critically ill patients, like in patients with acute respiratory distress syndrome, in order to optimize ventilator settings with the aim to reduce ventilator-induced lung injury [6].

Recent results suggest that intraoperative ventilation also is a risk factor for ventilator-induced lung injury [7,8]. Thus, intraoperative EIT monitoring of tidal volume distribution could be a tool to establish lung-protective ventilation, especially in patients at increased risk, such as for patients undergoing major open upper abdominal surgery. However, so far EIT has not been used during major open upper abdominal surgery due to several challenges, including interference with the surgical field, the long duration and electrocautery.

Therefore, we conducted a pilot-study throughout major open upper abdominal surgery and tested whether EIT is feasible and monitoring of ventilation distribution is possible.

## Methods

This study was approved by the local ethics committee (Ethikkommission der Medizinischen Fakultät, Heinrich-Heine-University Düsseldorf, Germany, study number 2974, Chairperson Prof. Lenard) on July 10^th^ 2008 and registered at ClinicalTrials.gov number 00795964 as part of a larger clinical trial [9]. With written informed consent, EIT was performed using the EIT Evaluation Kit 2 (Dräger Medical GmbH, Lübeck, Germany) with a 32×32 pixel resolution (1024 pixel per image) in consecutive patients scheduled for major open upper abdominal surgery of at least 3 hours duration.

### Study protocol and EIT

Prior to anesthesia induction, after placement of an epidural catheter at level T_7_-T_12_, the position of the 16 electrode silicone belt was determined in cooperation with the attending surgeon. Belt position was intended as caudal as possible, but high enough to prevent from interference with the surgical field. Accordingly, patient’s thoracic circumference was measured at that level and adequate belt size was chosen. Final belt position was documented according to the corresponding intercostal space at the mid axillary line and the neutral electrode was placed in the clavicular region. Skin contact was enhanced with electrode gel. Then, patients were positioned supine and asked to report inconveniences resulting from the belt.

Anesthesia was induced with thiopental (4-5 mg/kg) and sufentanil (0.4 μg/kg) and tracheal intubation was facilitated using cis-atracurium (0.2 mg/kg). Anesthesia was maintained with sevoflurane in air and 50% oxygen. Muscle relaxation was monitored by train-of-four ratio and repetitive relaxant boli were administered as deemed necessary by the physician in charge. The Zeus anesthesia system (Dräger Medical GmbH, Germany) was used for mechanical ventilation and positive end-expiratory pressure was set to 5 mbar (0.5 kPa). Ventilation mode was volume-controlled with auto-flow mode. According to the study protocol of the surrounding clinical trial [9], tidal volume was set to either 6 or 12 ml per kg of predicted body weight with an inspiratory-expiratory ratio of 1:2. Breathing frequency was adjusted to maintain end-expiratory CO_2_ at 35-40 mmHg (4.7-5.3 kPa).

#### EIT measurements

1) “Pre-Induction”: Prior to intubation patients were prompted to breathe steadily and calmly and one minute of spontaneous breathing was recorded. 2) After anesthesia induction, EIT measurements were repeated at the following time points: immediately after intubation (“Intubation”), shortly before skin incision and then every 60 minutes throughout surgery. To prevent risks from electrocautery, the belt was unplugged from the device after each measurement. Measurements were taken in agreement with the surgeon when no cautery was necessary. 3) A “Pre-Extubation” measurement was recorded shortly before the end of anesthesia while the patient was still on mechanical ventilation. 4) A last measurement was done after extubation in the post anesthesia care unit (“PACU”) during spontaneous breathing.

Patients were visited for 5 days post-surgery and investigated for skin lesions or burns.

### Feasibility

To assess the feasibility, we evaluated the number of patients and the intended measurements and compared it to the number of impaired or impossible measurements due to cauterization, reduced electrode skin contact or any other problems.

The performed measurements were then checked for validity: The EIT Evaluation Kit 2 can compensate for loss of contact of one skin electrode. Hence, EIT measurements were defined as technically valid when at least 15 electrodes provided sufficient skin contact, i.e. <301 Ω of electrode resistance. Additionally, we performed a visual inspection of recordings and requested an image quality high enough to allow identification of continuous breathing cycles with a stable end-tidal baseline for at least 30 seconds.

### Functional EIT data analysis

The raw EIT signal was analyzed with Dräger EIT data review software, Dräger Medical GmbH, Germany. First, a 50 per minute low pass filter was applied to separate impedance changes due to heart activity. For every breathing cycle in the recording period, a tidal image was created as a subtraction of end-expiratory from end-inspiratory impedances. Tidal images within the recording period were then averaged to generate a minute image. Data were then exported as ASCII files for further analysis.

### Tidal volume distribution throughout major open upper abdominal surgery

We analyzed our data for two parameters of tidal volume distribution: 1) ventilated lung area and 2) the center of ventilation index. Both parameters were compared before and after intubation as well as extubation, i.e. “Pre-Induction” versus “Intubation” and “Pre-Extubation” versus “PACU” measurements.

### Total and dorsal ventilated lung area

Ventilated lung area was assessed by distinguishing pixels that participated in impedance changes from those that did not. According to previous studies, a threshold of 20% of the maximum variation in the image was defined. The number of pixels with variations above this threshold were counted as total ventilated lung area [10,11]. Regions of interest were defined as horizontal, geometric halves of the EIT minute image and ventilated lung area in the dorsal half of the image was evaluated.

### Center of ventilation index

Center of ventilation index was calculated as previously defined [12,13]: Two-dimensional, i.e. antero-posterior, distribution of ventilation was quantified by addition of relative impedance changes per row of the EIT minute image. Then, the weighted average of the resulting antero-posterior histogram in % was calculated with values below 50% indicating a more ventral center of ventilation. Values above 50% indicate a more dorsal tidal volume distribution.

### Statistics

Normally distributed data are presented as mean ± standard deviation. K-S Testing revealed that ventilated lung area and center of ventilation was not normally distributed at a few individual time points. Thus, these data are presented as box plots. For statistical comparison between “Pre-Induction” versus “Intubation” and “Pre-Extubation” versus “PACU” values for ventilated lung area and center of ventilation index the Wilcoxon signed rank test was used. All calculations were performed with STATA 10.0, Statacorp, Texas, USA*.* A p <0.05 was considered statistically significant. Mean values of the center of ventilation index during spontaneous breathing prior to anesthesia of approximately 48% and a decrease by 7 ± 7% during controlled mechanical ventilation, indicating the magnitude of the ventral shift, have been reported [10]. A sample size calculation based on these values revealed that 10 subjects would be sufficient to show statistically significant differences with alpha of 0.05 and 80% power.

## Results

Intraoperative use of EIT was initiated in 14 consecutive patients. Patient characteristics, type of surgery and retractors, localization of incisions and EIT belt positions and sizes are displayed in Table 1. Median anesthesia duration was 8.2 hours, ranging from 4.6 to 13.75 hours. Prior to induction, all patients tolerated the silicone belt well and none expressed discomfort. Muscle relaxant was used for intubation in all patients and at least repeated once in 9 out of 14 patients (64%) throughout surgery. As part of the surrounding clinical trial, 7 patients were ventilated with a tidal volume of 6 ml per kg predicted body weight and 7 patients received 12 ml per kg predicted body weight. Tidal volumes remained unchanged throughout the whole examination period.

**Table 1:** Patients, type of surgery and intraoperative EIT belt positions in major open upper abdominal surgery

	
Number of patients	14
Female	4 (29%)
Age	66 ± 10
Height (cm)	175 ± 9
Body Mass Index (kg m^−2^)	25 ± 5
Thoracic epidural anesthesia	13 (93%)
Type of surgery	
Liver resection	5 (36%)
Whipple’s operation	5 (36%)
Gastrectomy	3 (21%)
Hemicolectomy	1 (7%)
Incision	
Transverse laparotomy	5 (36%)
Transverse laparotomy with upper midline laparotomy	8 (57%)
Midline laparotomy	1 (7%)
Use of Retractors	12 (86%)
Rib retractor (Rochard)	11 (79%)
Bookwalter Retractor	1 (7%)
Belt position	
Intercostal space II	4 (29%)
Intercostal space III	3 (21%)
Intercostal space IV	7 (50%)
Thorax circumference (cm)	104 ± 11
Belt size (M/L/XL/XXL)^*a*^	5/5/2/2 (36/36/14/14)

Values are total numbers and percentage in parenthesis or mean ± standard deviation. ^a^Belt sizes were selected according to measured thorax circumference: M (90-100 cm), L (100-110 cm), XL (110-120 cm) and XXL (120-130 cm).

### Feasibility

**Figure 1:**
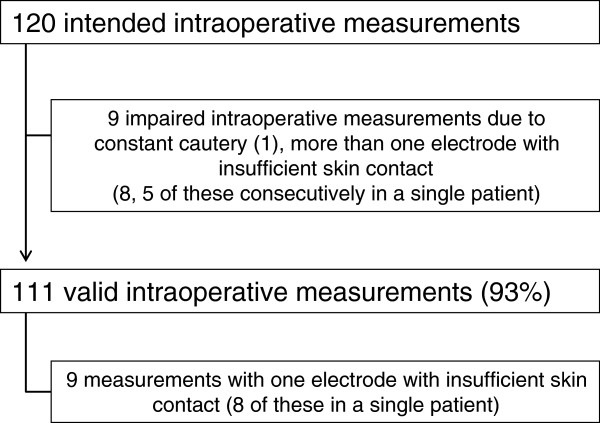
**Feasibility of intraoperative EIT measurements.** The majority of intraoperative measurements were feasible as planned.

Due to the individual duration of surgery, a maximum of 120 intraoperative measurements would have been achievable in our 14 patients, ranging from 5 to 15 measurements per patient. The majority of measurements (93%) were successfully performed and provided valid images (Figure 1).

Specifically, in 9 of 14 patients (64%) all intraoperative EIT measurements were feasible as planned. In one patient, the initial EIT belt appeared to be too large after anesthesia induction, which resulted in compromised electrode skin contact. Therefore, the belt was switched to a smaller size after intubation and intraoperative measurements were performed uneventfully. In contrast, in one out of 14 patients (7%) EIT was not feasible throughout liver resection of 5 hours duration. Despite all efforts, we were not successful in establishing secure electrode skin contact for two dorsal electrodes in this slim female. In 3 patients EIT measurements were impaired at single time points only. Additionally, in one male patient undergoing liver resection, one electrode did not have sufficient skin contact throughout surgery, but images were valid. Thus, in 64% of cases EIT was feasible, in 29% smaller problems occurred and in 7% EIT was not possible throughout open upper abdominal surgery.

Postoperatively, four patients remained on mechanical ventilation and thus no “PACU” measurements are available. During postoperative visits, no burns or other skin lesions were detected that might have been caused by the EIT belt.

### Tidal volume distribution

**Figure 2:**
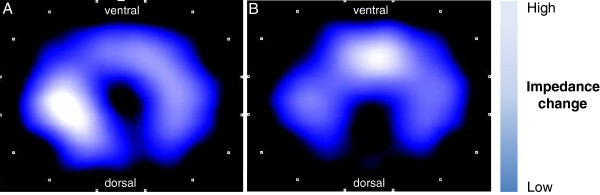
**EIT minute images before and after start of mechanical ventilation using a high belt position.** Two representative EIT images of a single patient: the blue silhouette shows the intra-thoracic gas content. The magnitude of impedance changes is color coded and ranges from dark blue, which indicates low changes to high white, which indicates high impedance changes. Panel **A**: prior to intubation (“Pre-Intubation”) the maximum impedance change occurs in the middle of the thorax. Panel **B**: after intubation and start of mechanical ventilation (“Intubation”) impedance changes are highest in ventral lung regions.

Figure 2 shows a representative example of minute images derived from the same patient comparing “Pre-Induction” and “Intubation” gas distribution using a high belt position. White coloring indicates high impedance changes, which shows where most of the tidal volume is distributed to. Visual inspection indicates a shift of tidal volume distribution to more ventral lung areas at the start of mechanical ventilation as compared to spontaneous breathing.

#### Total ventilated lung area

**Figure 3:**
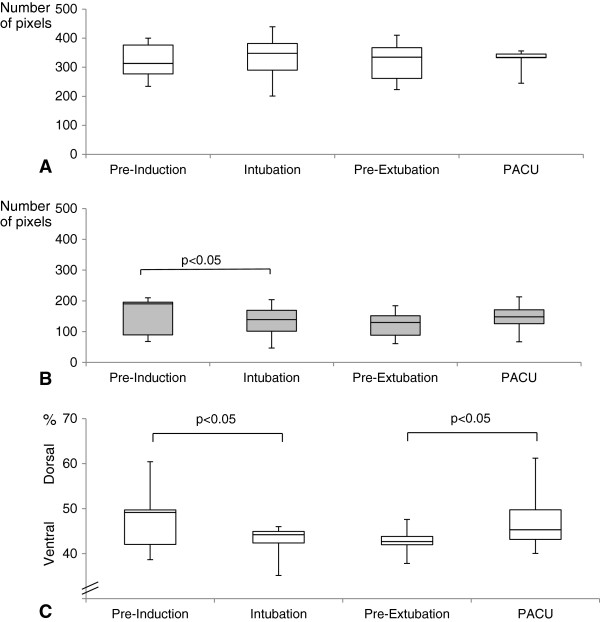
**Tidal volume distribution throughout major open upper abdominal surgery. A)** Total ventilated lung area, **B)** Dorsal ventilated lung area **C)** Center of ventilation index. Boxplots display median, interquartile range, minimum and maximum values. Statistically significant differences are indicated. “Pre-Induction” = spontaneous breathing prior to anesthesia induction, “Intubation” = mechanical ventilation shortly after intubation, “Pre-Extubation” = mechanical ventilation at the end of surgery shortly prior to extubation, PACU=“Postanesthesia care unit”, spontaneous breathing after extubation. Analysis of tidal volume distribution at “Intubation” was not possible in three patients due to insufficient skin contact with more than one electrode, thus measurement of 11 patients were available at this time point. “PACU” measurements were unavailable from four patients who remained on mechanical ventilation and were considered invalid in one additional case, resulting in data from 9 patients.

Total ventilated lung area did not change significantly with start of mechanical ventilation and remained comparable until spontaneous breathing was restored (Figure 3A). In contrast, dorsal ventilated lung area decreased significantly with intubation (Figure 3B, p = 0.028). Although the values increased again after extubation, this difference between “Pre-Extubation” and “PACU” was not statistically significant (p = 0.477).

#### Center of ventilation index

The center of ventilation index showed a significant shift towards more ventral ventilation after intubation (p = 0.027), which remained unchanged throughout surgery. After extubation this effect was reversed (p = 0.038) (Figure 3C).

## Discussion

This is the first study to show that EIT usage throughout major open upper abdominal surgery is feasible. In the majority of our patients consecutive EIT images were obtained as planned throughout long lasting procedures ranging from 5 to 13 hours duration. However, in individual cases the appropriate skin contact of electrodes constituted a major obstacle. As a consequence, we experienced one case in which we could not obtain consecutive EIT images throughout surgery. Despite the use of contact gel, sufficient skin contact was not always easily facilitated. Besides problems with epidural catheter patches that interfered with dorsal electrodes, anterior electrode skin contact in the inter-mammarian region was impaired in obese or female patients with large breast. Those problems had to be solved before surgical skin disinfection, since belt position can hardly be altered during surgery. However, with careful preparation, valid measurements were acquired in the majority (93%) of the potential measurements. Still EIT imaging might be hindered in individual cases. Reports on intraoperative use of EIT and actual measurements throughout surgery are limited to two studies so far. One reports on orthopedic patients undergoing leg surgery of one and a half hours duration [13]. The other studied patients undergoing laparoscopic gastric bypass surgery of approximately two hours duration [14]. In contrast, Erlandson et al. did not use EIT throughout laparoscopic gastric bypass surgery as to avoid problems from electrocautery and consequently applied it before and after surgery [15]. In two other trials, EIT imaging was restricted to anesthesia induction in pulmonary and cardiac surgery patients because of interference with the surgical field [16,17]. Interestingly, the two studies in which EIT was employed intraoperatively do not report any technical difficulties. Although, Radke et al. mentions loss of one measurement due to “device malfunction caused by interference from the electrocautery” [13], the other one does not mention technical considerations and both do not state preventive measures for phases of electrocautery [14].

In our trial presented here we considered both problems, potential interference with the surgical field and danger of burns from electrocautery. First, to adjust EIT application to the open surgical field in the upper abdomen, we applied the belt in close cooperation with the surgeon. Consequently, it was positioned higher than usual. When EIT is applied conventionally, the belt is placed at the level of the sixth intercostal space [13]. In our cases of open upper abdominal surgery, the belt was placed as caudal as possible which was under the arm-pit at the second to 4^th^ intercostal space. In this position a more cranial lung area is depicted by the EIT images. Bikker et al. recently compared EIT images in postoperative patients derived from the 6^th^ or 3^rd^ intercostal space. They found that tidal ventilation distribution can differ between the two levels [18]. On the other hand, during open upper abdominal surgery a cranial shift of lung and diaphragm occurs, which may, at least in part, counterbalance the high belt position [19]. Second, EIT is impossible during electrocautery, because it results in invalid measurements. But even more important, the electrical current may be transduced via the EIT electrodes, which potentially causes skin burns. To prevent this major hazard, we disconnected the EIT belt from the device and reconnected it only prior to each measurement, as advised by the manufacturer. Although a necessary premise, this is somewhat inconvenient for routine use of intraoperative EIT. Disconnecting the belt from the device results in loss of end tidal baseline and thus the absolute impedance changes are incomparable between time points. However, relative indices such as the center of ventilation index remain unaffected. In long measurement series, baseline would have to be readjusted in any case to compensate for changes in electrode impedance, such as skin conductivity altered by sweating. Under these precautions, we detected no adverse effects such as skin lesions or burns. In summary, EIT was feasible under special premises most cases of major open upper abdominal surgery.

Measurements during major open abdominal surgery are only possible using a high belt position and we detected the typical shift of tidal volume distribution towards ventral regions after intubation [10,13,14]. The center of ventilation index increased significantly with start of mechanical ventilation. Interestingly, this effect was reversed after extubation, despite the long duration of surgery and the repetitive use of muscle relaxant. Of note, all patients received a manual recruitment maneuver prior to extubation. Thus, our findings are comparable to those of Radke et al., who studied the center of ventilation index in patients with shorter peripheral operations undergoing controlled mechanical ventilation without muscle relaxant [13].

We also found that the total ventilated lung area remained unaffected during mechanical ventilation of substantial duration. However, it decreased significantly in the dorsal region of interest. Although EIT cannot directly visualize atelectasis, this indicates that dorsal atelectases can also be measured with a high belt position. Hence, anesthesia-induced tidal volume shifts can be monitored during long lasting open upper abdominal surgery with the belt in this position. Interestingly, these differences in tidal volume distribution are a matter of active or passive diaphragm movement. During spontaneous breathing in supine position, the dependent dorsal part of the diaphragm has the highest contraction. This is due to the higher mobility of the dorsal and lateral parts of the diaphragm than of the ventral part, which is fixed to the sternum. With muscle relaxation and mechanical ventilation the intra-abdominal pressure causes a cephalad shift of the diaphragm and the ventilation pressure leads to tidal volume distribution into non-dependent ventral areas where the surrounding pressure is least [20]. Accordingly, recent EIT results suggest, tidal volume is more homogeneously distributed in patients with Acute Respiratory Distress Syndrom undergoing pressure support ventilation [5].

### Limitations

This was a pilot study in a limited number of patients undergoing upper abdominal surgery under very cautious premises. Although there were no complications attributed to EIT measurements (e.g. skin lesions or burns), general safety of this method during upper abdominal surgery can only be assessed with much larger trials.

In this pilot-study, we did not perform interventions to alter tidal volume distribution or optimize ventilatory settings, because we did not know, whether imaging would be feasible at all. However, beneficial effects will only derive from EIT use in open upper abdominal surgery if individual ventilation is optimized.

Patients were ventilated with two different tidal volume settings (6 or 12ml per kg predicted body weight). Comparison of tidal volume distribution between these groups would have been very interesting, but was not performed due to the limited sample size. For the same reason, we did not analyze individual courses of intraoperative consecutive measurements. However, with a PEEP of 5 mbar in both groups, low tidal volume ventilation was associated with significantly lower oxygenation and more atelectasis in the surrounding larger trial [9]. Thus, in patients undergoing low tidal volume ventilation, a decreased ventilated lung area could trigger the use of appropriate PEEP levels and/or recruitment maneuvers to prevent or treat atelectasis. In contrast, high tidal volume ventilation increases the risk of hyperinflation in independent lung areas, thereby increasing the risk of ventilator-induced lung injury. The center of ventilation index could indicate a more ventral distribution in patients with hyperinflation of the independent lungs. We cannot rule out, that the different settings influenced our findings. Therefore, larger trials are necessary to show if EIT is useful to guide such intraoperative optimization of ventilatory settings.

## Conclusions

Under cautious premises and with a high belt position EIT is feasible and monitoring of ventilation distribution is possible in patients undergoing major open upper abdominal surgery. These patients are at high risk for postoperative pulmonary impairment and ventilator induced lung injury. Thus, our study is the basis for further interventional trials in this setting in order to optimize ventilatory management.

## Competing interests

The authors declare that they have no competing interests. This research was performed with institutional funding.

## Authors’ contributions

MSS was involved in conduct of the study, data collection and analysis and manuscript preparation. VW was involved in conduct of the study, data collection and helped with the final analysis. BB and US were involved in conduct of the study and data collection. PK and MB were involved in design of the study and manuscript preparation. TAT was involved in design of the study, analysis and manuscript preparation. All authors approved the final manuscript.

## Pre-publication history

The pre-publication history for this paper can be accessed here:

http://www.biomedcentral.com/1471-2253/14/51/prepub
